# The involvement of rTPJ in intention attribution during social decision making: A TMS study

**DOI:** 10.3758/s13415-024-01188-7

**Published:** 2024-04-30

**Authors:** Francesco Panico, Antonella Ferrara, Laura Sagliano, Luigi Trojano

**Affiliations:** University of Campania “Luigi Vanvitelli, Viale Ellittico 31, 81100 Caserta, Italy

**Keywords:** Social decision making, Temporoparietal junction, Transcranial magnetic stimulation, Ultimatum Game

## Abstract

The mini-Ultimatum Game (mini-UG) is a bargaining game used to assess the reactions of a responder to unfair offers made by a proposer under different intentionality conditions. Previous studies employing this task showed the activation of responders’ right temporoparietal junction (rTPJ), which could be related to its involvement in judgments of intentionality. To verify this hypothesis, in the present study we applied online transcranial magnetic stimulation (TMS) over the rTPJ in responders during the mini-UG, in which we manipulated intention attribution implicitly. A cover story was employed to induce participants to believe they were interacting with another agent. We expected that interfering with the rTPJ could affect the ability of responders to assume proposers’ perspective, producing higher rates of rejections of unfair offers when offers are perceived as independent from responders’ intentionality to inequality. Twenty-six healthy women voluntarily participated in the study. In the mini-UG, an unfair distribution of the proposer (8/2 offer) was pitted against one of three alternative offers: fair-alternative (5/5), no-alternative (8/2), hyperfair-alternative (2/8). During the task, a train of TMS pulses was delivered at proposers’ offer presentation in blocks of active (rTPJ) or control (Vertex) stimulation according to an ABAB design. As expected, findings showed that rejection of the no-alternative offers was higher under TMS stimulation of the rTPJ compared with the control TMS. This effect was modulated by the degree of trustworthiness in the cover story. These data contribute defining the mechanisms and brain areas underpinning social decision making as assessed by bargaining tasks.

**Supplementary Information:**

The online version contains supplementary material available at 10.3758/s13415-024-01188-7.

## Introduction

Social decision making concerns taking choices in a social context, thereby considering how decisions are shaped by the intentions, opinions, and preferences of the other agents involved (Báez-Mendoza et al., 2021). Starting from the early work by Simon (1967), in the last decades the normative model of rational choice has been renovated to include cognitive, contextual, emotional, and social features (for reviews see: Lerner et al., 2015; Rilling & Sanfey, 2011). At the same time, numerous games have been developed to target decision making in experimental contexts reproducing situations in which individuals must make decisions, thus allowing to understand human choices in complex social issues (Van Dijk & De Dreu, 2021). A well-established task in this context is the Ultimatum Game (UG; Güth et al., 1982), where a player (responder) is faced with a monetary offer from another player (proposer) who has to share part of the stake with the responder without any constraint. A variation of this task limits the possible proposer’s choices to just two alternatives, which are known to the responder as well (mini-UG; Falk et al., 2003). One of the alternatives is always an unfair division of the stake (*unfair offers*), whereas the second alternative can be fair (*fair offer*s) or unfair again (meaning the proposer has *no alternative* but to make an unfair offer). This variation of the task, compared with the traditional one, allows to evaluate social decision making under different intentionality constraints, because both responders and proposers must mentalize the intentions of the other player when proposing or evaluating a given offer. Crucially, this task allows to disentangle a general aversion to unfairness, namely rejection of unfair offers independently from the context, from a more specific process of attribution of intentionality to the partner, namely rejection of unfair offers exclusively when the proposer has a better alternative but deciding to propose the unfair one. Previous studies using this task showed that responders are more inclined to accept unfair offers in the *no alternative* condition compared with the *unfair offers* condition (Falk et al., 2003; Güroǧlu et al., 2010). In the interpretation of this evidence, Güroǧlu et al. (2010) reasoned that evaluation of *no alternative* offers would require the highest level of intentionality understanding, because rejecting an unfair offer, which is disadvantageous from a strict rational perspective, cannot be immediately justified. The result of their study showed that responders more often rejected unfair offers when the proposer also could have selected a fair offer, suggesting that participants valued fairness even when this occurred at the cost of their own benefit (Falk et al., 2003; Fehr & Schmidt, 1999). Crucially Güroǧlu et al. (2010) found that rejection rates were significantly reduced when proposers had *no alternative* but to offer an unfair division.

Many studies have investigated the contribution of cortical and subcortical regions to choices in the social context (Rilling & Sanfey, 2011; Rudebeck et al., 2008), as well as the brain circuits and networks connecting these areas (Báez-Mendoza et al., 2021). As reviewed by Rilling and Sankey (2011), critical areas include the subdivisions of the prefrontal cortex (involved in evaluating long-term benefits and in the cognitive effort to control for impulses), the anterior cingulate cortex and the striatum (involved in reward mechanisms and in reaction to social norm violations), the anterior insula (involved in inequity aversion and empathy), and the amygdala (involved in the emotional responses associated with the decision). Moreover, recent studies outlined the contribution of the temporoparietal junction (TPJ), mainly in the right hemisphere (rTPJ), which would be involved in the attribution of intentions to others and in the process of mentalizing (Hao et al., 2023; Langenbach et al., 2022; Lee & Seo, 2016; Saxe & Wexler, 2005). Other studies also described an involvement of the TPJ in fairness and moral judgements (Jeurissen et al., 2014; Ye et al., 2015; Young et al., 2010) and in the network underlying rational choices (Kahnt & Tobler, 2013; Ni & Li, 2021; Zhang et al., 2017). All these abilities may be activated during social decision making, although the attempt to comprehend their role can be challenging. In the above-mentioned study using the mini-UG, Güroǧlu et al. (2010) found that rejection of unfair offers in the *no alternative* condition resulted in enhanced activity of rTPJ, which was interpreted in terms of higher mentalizing demands required in social decision making when rejection could not be readily justified. However, this intepretation remains speculative given the correlational nature of neuroimaging methods, and the specific contribution of the rTPJ in the process of social decision making remains open to debate.

Several previous studies investigated the involvement of the TPJ in social decision making. Li et al. (2020) delivered electric stimulation over the TPJ during the dictator game and found anodal stimulation to increase charitable giving and egalitarianism. However, Gaesser et al. (2019) did not observe effects on willingness to help following imagined or remembered prosocial episodes after magnetic stimulation over the TPJ. Moreover, neuroimaging studies have proposed a role of the TPJ in generous choices. Park et al. (2017) found that participants receiving instruction to spend money on others during a 4-week period, compared with control participants committed to spend money on themselves, eventually made more generous choices during a decision making task together with an enhanced TPJ activity. Interestingly, experimental participants reported an increase in their subjective happiness ratings associated with a modulation of the connectivity between the TPJ and the ventral striatum (Park et al., 2017). Similarly, Strombach et al. (2015) described an increase in TPJ activation during experimental conditions were participants decided to overcome the egoistic choice to maximise their own gains in place of more generous decisions, suggesting a mechanism of upregulation between the TPJ and the ventromedial prefrontal cortex integrating other-regarding preferences into own-reward value representation (Strombach et al., 2015). Taken together, these data seem to support a central and multifaceted role of the TPJ in a heterogeneous range of social behaviors, which also involves taking choices during social transactions. Nonetheless, because of the inconsistencies among studies, the specific processes in which the TPJ is involved are far from being fully comprehended. Clarifying the contribution of the TPJ in social decision making would allow refining the models on the mechanisms and processes underlying social cognition and social decision making as assessed by bargaining tasks. Specifically, it is worth investigating whether this region is generally involved in the processes that evaluate fairness and equity during social interaction or in a more specific mechanism of intention attribution.

To tackle this issue, we implemented an experimental paradigm by using online transcranial magnetic stimulation (TMS) over the rTPJ in responders during the mini-UG, where the different possible offers allowed to manipulate intention attribution implicitly. This made possible to interfere with the activity of the rTPJ during the decision making process in responders and to obtain cues on the involvement of this region in evaluating the different offers received. On the basis of the previous literature (Güroǧlu et al., 2010; Jeurissen et al., 2014; Saxe & Wexler, 2005; Zhang et al., 2017), three possible scenarios were plausible. First, if the rTPJ contributes to the processes of decision making, interfering with its activity would affect the whole process of bargains’ evaluation, thus producing higher rejections in *fair*, *unfair*, and *no alternative* offers. Second, if the rTPJ is involved in the mechanisms of moral evaluation during social interactions, we could expect an increase of rejections of both *unfair* and *no alternative* offers, leading to a refusal of all proposals evaluated as unfair independently from the context. Third, and crucially, if the rTPJ is involved in appraising others’ intentions in a specific context of social decision making, a clear-cut selective increase in the rejections of *no alternative* offers would be expected, meaning that the responders are impaired in mentalizing the other has no better bargain to propose.

## Materials and method

### Participants and experimental design

A single session, within-subject TMS experimental design was adopted. The sample size was estimated based on a priori power analysis conducted with G*Power 3 (Faul et al., 2007), suggesting a sample size of 19 participants (6 measurements, effect size of 0.25, power of 0.80, and alpha of 0.05). As the procedure involved a cover story and as the belief in the story was a prerequisite for proper assessment of social decision making, we assessed the extent to which participants believed in the cover story at the end of each session (a complete description of the procedure is reported below). Recruitment and data collection continued until the number of 19 “believers” was reached. Moreover, to avoid possible confounding effects related to gender differences in decision making (Karmarkar, 2023; Sarlo et al., 2013; van den Bos et al., 2013; Vanutelli et al., 2020), we planned to include only women in the present study. Thus, we enrolled 26 right-handed female university students (age range = 19–27; average age = 20.81, SD = 1.88) who voluntarily participated in the study. Participants had normal or corrected-to-normal vision, had no history of neurological or psychiatric disease, and were naïve to the purposes of the study.

The participants completed an interview confirming eligibility to take part in a TMS study and gave their written, informed consent to take part in the experiment. The procedure was in agreement with 1975 Helsinki Declaration and was approved by the Ethic Committee of the Department of Psychology (n. 18/2023).

### Cover story

The participants were informed that the goal of the experiment was to evaluate decision-making processes during social interactions and that they would play a game with a university student from another institution via a web application. The game consisted of sharing some coins with the other player, which could be converted at the end of the game to obtain a gadget of equivalent value (in truth all participants received a gadget of the same value at the end of the experiment). This was made to ensure participants’ engagement in the social interaction. No anticipation on the kind of the gadget or its value was provided. After drawing from a box, all participants were told that they were attributed to the role of responder, and that they had to accept or refuse the offers made by the other player. To increase the credibility of the cover story and to maximize the feeling of being in interaction with another player, the participants were required to prepare a brief presentation clip to be sent to the second player and watched a (standard) presentation clip of the (fake) proposer. In fact, there was no second player, and the participants always played with a computer with predefined pseudorandomized offers.

### The mini-ultimatum game

The task used in this experiment was adapted from that devised by Güroǧlu et al. (2010). All participants took the role of responder during the Mini-Ultimatum Game (mini-UG) and played with the same (fake) partner throughout the task. The task involved three conditions, where an unfair distribution of the proposer (8 coins for the proposer/2 coins for the responder; unfair offer) was pitted against three alternative offers, namely 5/5 offer (fair-alternative), 8/2 offer (no-alternative), 2/8 offer (hyperfair-alternative). It included four blocks of 34 trials lasting approximatively 5 min each, comprising ten unfair trials, ten fair trials, ten no-alternative trials, and four hyperfair trials. The total number of trials was 136; the overall number of trials in the present study was reduced with respect to that original version of the task (n = 168 in Güroǧlu et al., 2010) to comply with technical requirements (coil heating following sustained use) and safety recommendations (Rossi et al., 2009) related to TMS. Hyperfair trials were included in our task just not to give the impression that responders were playing an entirely unfair game, with exclusively unfair or equal offers and no offers favoring their gains. However, hyperfair trials were not considered in the analysis, thus the number of these offers was reduced with respect to Güroǧlu et al. (2010).

**Fig. 1:**
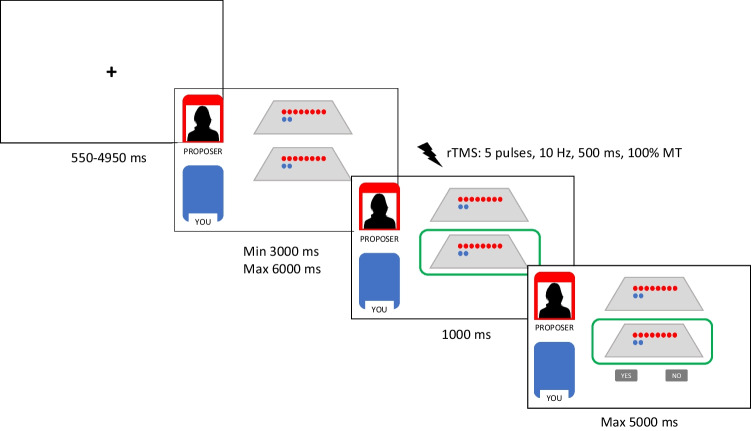
Trial sequence of the mini-UG (an example of no alternative offer is described in the figure; rTMS = repetitive transcranial magnetic stimulation; MT = individual’s Motor Threshold).

Figure 1 exemplifies a trial sequence for the task. Each trial started with a fixation cross (random duration ranging 550–4950 ms), followed by a screen displaying the two possible alternatives among which the proposer could choose; a variable time was used (ranging 3000–6000 ms) to maximize the belief of their interaction with a human proposer making an offer. Then, the offer was revealed to the responder followed by the response buttons (accept vs. refuse, 1000 ms). Participants had to press the button “n” or “m” by using the index and middle fingers of the right hand to express their decision (response keys counterbalanced across participants). The maximum response time was set at 5000 ms, and then the next trial was displayed. No feedback was given to the participants during the task. Trial randomization and presentation, online TMS administration, as well as data collection were performed using MATLAB software package (version 2021a, MathWorks, Natick, MA) with the Psychophysics Toolbox extensions (Brainard, 1997; Pelli, 1997). Participants’ responses and reaction times (RTs) were recorded.

### Repetitive transcranial magnetic stimulation (rTMS)

Online trains of repetitive transcranial magnetic stimulation (rTMS) (5 pulses, 10 Hz; duration: 500 ms) were delivered on the onset of the offer display by means of a 70-mm-diameter figure-of-eight coil connected to a Magstim Rapid 2 stimulator (Magstim Company) producing a maximum output of 1.2 T at the coil surface (type of output: biphasic; pulse width: 400 μs). Stimulation intensity was fixed at 100% of the participants’ motor threshold (MT, mean MT = 53.42, SD = 2.67). The rTPJ and the corresponding stimulation site on the participants’ scalp was localized by means of Softaxic Optic (EMS) neuronavigation system. The brain coordinates for this region (x = 63, y = −48, z = 33) were based on a previous neuroimaging study using the mini-UG task (Güroǧlu et al. 2010). Neuronavigation was performed on estimated-MRI stereotaxic templates of participants’ brains based on a sampling of 24 scalp points digitized by means of a Polaris Vicra (Northern Digital) digitizer. In the control condition, rTMS was delivered to the Vertex which corresponded to Cz according to the 10–20 EEG system for electrode placement. This control region was defined as the crossing of the midline between the inion and the nasion, and the midline between the left and the right preauricular points, and previous studies support its use as an appropriate control site in TMS investigations (Jung et al., 2016). During stimulation, coil positioning on the stimulation sites was monitored online by means of the neuronavigator system for the entire experimental session (maximum accepted distance from the site 3 mm; maximum accepted radius 5 degrees); in all conditions, a mechanical arm attached to a tripod hold the coil. TMS trains were delivered at proposers’ offer presentation and rTPJ and vertex stimulation were delivered in alternate blocks, corresponding to the four task blocks described above, using a ABAB design (counterbalanced across participants).

### Manipulation check

An ad hoc post-experimental questionnaire was administered at the end of the experiment to assess the degree to which the participants believed in the cover story (see Supplementary Materials). To increase the face validity of the questionnaire, it included some general questions targeting how much they felt energic, tense, sleepy, and pleased about the interaction with the other participant, together with the target question about to what extent they had the impression of being in interaction with another person during the game. The participants provided their responses on a five-point Likert scale (1 = “not at all”, 5 = “completely”). All participants were debriefed and received extensive explanation on the purpose of the study at the end of the experiment and on the need to use a cover story. Moreover, all participants were given a gadget (i.e., cosmetic products) as a token of appreciation for their participation.

### Data analysis

As specified above, to believe in the cover story was relevant for our research protocol assessing social decision making. Therefore, our primary analyses were based on the results of participants who believed they were interacting with another person (the “believers,” who provided 3–5 ratings on the Likert scale for the above target question). The responses of the participants who did not believe in the cover story (1–2 ratings on the Likert scale for the target question) were not included in the primary analyses. Rate of rejections and mean reaction times (RTs) were calculated for each condition. Normality of the dependent variables was assessed by the Shapiro-Wilk test and graphical (Q-Q plots) data (Ghasemi & Zahediasl, 2012; Mishra et al., 2019). We then compared the number of rejections and RTs for the type of offer (alt-fair, alt-unfair, no-alt) and stimulation (rTPJ-TMS and Vertex-TMS) as within group factors. For this primary analysis, we planned to calculate Bayesian Factors to quantify the strength of evidence in favor of a difference in the performance for the three offer types during the two stimulation conditions (BF; van Doorn et al., 2020). The BF were interpreted according to Quintana and Williams (2018). To control for any order effect of stimulation on performance, we compared rejections for fair, unfair, and no-alternative trials during Vertex-TMS in participants receiving the Vertex-TMS as the second block (N = 11), with participants receiving Vertex-TMS as the first block (N = 8).

Moreover, because we also collected data from participants who did not believe in the story, we had the opportunity of running secondary analyses considering the engagement in the cover story as assessed by the post-experimental questionnaire. We could assess whether the belief in the cover story (namely ascribing the offers to a real person’s choice) affected participants responses. For this purpose, we compared the number of rejections and RTs for the type of offer (alt-fair, alt-unfair, no-alt) and stimulation (rTPJ-TMS and Vertex-TMS) in the group of “believers” with those of “non-believers” (the participants who did not felt of being in interaction with another person during the game; 1–2 ratings for the target question).

To confirm the possible specific effect of the belief of being in interaction with another person as an explanatory factor, we also planned to look for associations between the degree of belief in the cover story (1–5 scale) and the number of rejections of each offer under rTPJ and vertex stimulation on the whole sample.

Post hoc comparisons were performed by Bonferroni-corrected tests, with level of significance set at *p* < 0.05.

## Results

### Number of rejections

Based on participants’ evaluation, we excluded seven participants who did not believe in the cover story (“nonbelievers”) for the main analyses. Thus, the final sample included 19 “believers” (average age = 20.52, SD = 1.54; average MT = 53.05, SD = 2.76).

Normality checks showed that the number of rejections was not normally distributed (S-W values ranging .29–.89; all *p* < .01), thus nonparametric, conservative methods of data analysis were implemented. To estimate effect sizes we followed Fritz at al. (2012) and computed Cramer’s V for nonparametric analysis of variance and r for paired and unpaired comparisons; after Fritz et al. (2012), effect size was considered small within .1–.3 range, moderate within .3–.5 range, and large above .5.

**Fig. 2:**
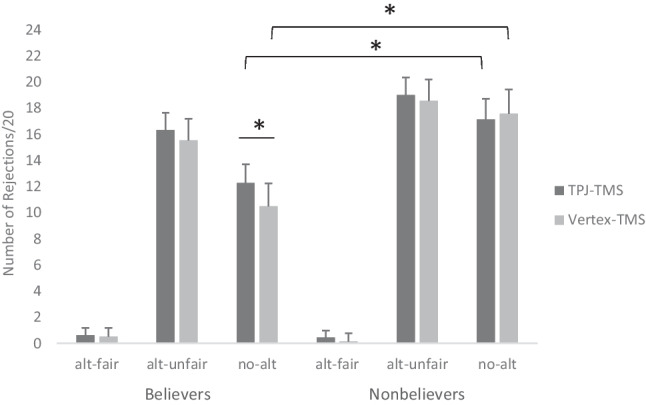
Results of nonparametric statistics on rejections in responders for the different offers received by the proposer. *Significant for *p* < .05

A Friedman Analysis of Variance, run to compare the number of rejections for the type of offer and stimulation, showed a significant difference in the number of rejections as a function of the offer and stimulation type [χ^2^(5) = 72.33, *p* < 0.001, Cramers’V = .87]. Paired Wilcoxon comparisons on the number of rejections showed that both under rTPJ-TMS and Vertex-TMS stimulation, rejections were higher for the alt-unfair trials compared with the alt-fair trials (Z = −3.86, *p* < .001, r = .88; Z = −3.75, *p* < .001, r = .86) and compared with the no-alt trials (rTPJ-TMS: Z = −3.11, *p* = .002, r = .71; Vertex-TMS: Z = −3.21, *p* = .001, r = .74). Moreover, the number of rejections was higher in the no-alt trials compared with the alt-fair trials in the two stimulation conditions (rTPJ-TMS: Z = −3.73, *p* < .001, r = .85; Vertex-TMS: Z = −3.30, *p* = .001, r = .76). Crucially, statistical analyses revealed increased rejections in the no-alt trials under rTPJ-TMS compared with the no-alt trials under Vertex-TMS (Z = −2.49, *p* = .01, r = .57; Fig. 2). Comparison of rejections for alt-fair (Z = −.42, *p* = .67; r = .09) and alt-unfair trials (Z = −1.27, *p* = .21, r = .29) in the two stimulation conditions was not statistically significant. To quantify the strength of evidence in favor of a difference on the number of rejections in the two stimulation conditions for the three offer types, we calculated Bayesian Wilcoxon signed-rank tests (van Doorn et al., 2020) and interpreted values of BF according to Quintana & Williams (2018). Results supported evidence in favor of the null hypothesis for alt-fair (BF10 = .27) and alt-unfair offers (BF10 = .46) during the two stimulation conditions, while they supported the alternative hypothesis for no-alt offers during rTPJ-TMS and Vertex-TMS (BF10 = 11.62; see Supplementary Materials). Mann-Whitney tests conducted on rejections for fair, unfair, and no-alternative trials during Vertex-TMS comparing participants receiving the Vertex-TMS as the second block with participants receiving Vertex-TMS as the first block resulted in no significant difference (see Table 4 of Supplementary Materials).

To explore how results were affected by the belief of engaging in an authentic social interaction, we performed post-hoc analyses comparing the subgroup of “believers” (n = 19) with the responses of participants who gave low ratings on the target question of the post-experimental questionnaire (“nonbelievers”: n = 7; average age = 21.57, SD = 2.57; average MT = 54.42, SD = 2.29). The comparisons of the number of rejections in these two subgroups for the different trials and stimulation conditions by means of Mann-Whitney test showed that rejections significantly differed in the two subgroups in the no-alt trials during both rTPJ-TMS (U = 101, *p* = .048, r = 0.39) and the Vertex-TMS (U = 105, *p* = .025, r = 0.44), with higher rates of rejections in nonbelievers compared with believers (Fig. 2). No other significant differences were found in the two groups (see Table 1 of the Supplementary Materials).

**Fig. 3:**
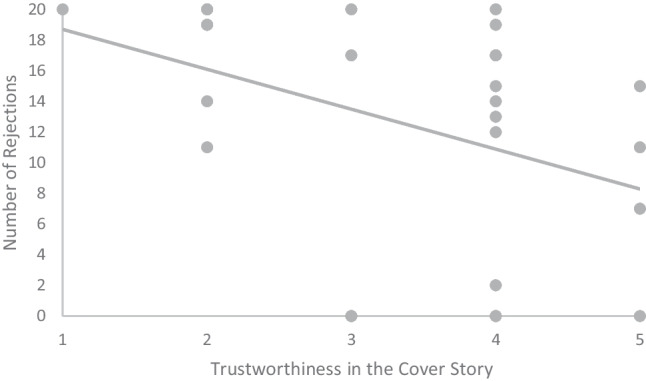
Association between number of rejections and degree of belief of engaging in authentic social interaction in responders under vertex stimulation.

Nonparametric Spearman correlations to look for associations between the degree of belief in the cover story (1–5 scale) and the number of rejections of each offer under rTPJ and vertex stimulation on the whole sample showed a significant negative correlation [r = −45, *p* = .02, n = 26] between ratings on the cover story and the number of rejections of no-alternative trials under vertex (not rTPJ) stimulation (Fig. 3). No other significant correlation was observed (see Table 2 of the Supplementary Materials).

### RTs

Normality check revealed that RTs did not approximate a normal distribution (S-W values ranging .79–.91; all *p* < .05). Therefore, nonparametric analyses also were run for RTs. The main analysis was run on data from the 19 participants who believed in the cover story (7 participants excluded).

A Friedman Analysis of Variance comparing RTs depending on the type of offer and stimulation condition yielded not significant results [χ^2^(5) = 9.11, *p* = .11, Cramers’V = .30].

As above, we also compared RTs in believers (n = 19) and nonbelievers (n = 7) on the different offer types and stimulation conditions by means of Mann-Whitney test. The analyses showed no significant differences in the two subgroups (see Table 3 of the Supplementary Materials). No effect related to the order of stimulation was observed on RTs (see Table 5 of Supplementary Materials).

## Discussion

The purpose of the present study was to contribute to the literature on the brain correlates underlying social decision making, focusing on the specific involvement of the rTPJ in a bargaining task under different intentionality conditions. For this purpose, a single session, within-subject TMS experimental design was adopted while participants completed the mini-UG task in the role of responders. To reproduce the mechanisms in action during social interaction, possibly mimicking real-life situations, a cover story was implemented to maximize participants’ feeling of being in a game involving another agent. The strength of belief in the cover story was controlled for.

The results demonstrated that under TMS over the rTPJ (compared with the Vertex), the participants who felt to be engaged in the social interaction provided an increased number of rejections in response to the no alternative offers compared with the fair and unfair offers. Moreover, our secondary analyses comparing the participants who believed in the cover story to the ones who did not detected in the latter subgroup an overall higher number of rejections for the no alternative offers.

### Increase in rejections of no alternative offers following rTPJ stimulation

The main results of the present study showed that rejection rates in responders reached the highest value in reaction to unfair offers. Indeed, rate of rejection of *unfair offers* was higher compared with *fair offers* and *no alternative offers*; *no alternative offers* were rejected more frequently compared with *fair offers*. These results are in line with data from previous studies using the same task (Falk et al., 2003; Güroǧlu et al., 2010) and allow to confirm successful implementation of the task in our experimental design.

Most importantly, we found that the number of rejections was higher during rTPJ-TMS compared with Vertex-TPJ specifically for the *no alternative offers*. Rejections of *fairs offers* and *unfair offers* was not affected by stimulation. This allows to discuss the specific role of the rTPJ during social decision making as assessed by the mini-UG. As described above, the modified version of the task used here (Falk et al., 2003) allows to target the behavior of the responders to specific offers received by the proposer under different intentionality conditions (Güroǧlu et al., 2010). More specifically, as argued by Güroǧlu et al. (2010), evaluation of *no alternative offers* would be associated with the highest intentionality demands, as the evaluation of these offers would require the ability to consider that the other player has no other alternative but to propose an unfair offer, thus prompting the responders to accept the unfair bargain. To better understand the role of the rTPJ in the mechanism of intention attribution, we interfered with the activity of this region and found that rTPJ stimulation increased rates of rejections in the *no-alternative* condition, thus confirming its involvement in the appraisal of these offers. Critically, no effect was observed when responders evaluated fair and unfair offers. The lack of any change in rejection rates for these latter offers following stimulation also allows to discuss the functions the rTPJ would *not* detain during the mini-UG. Indeed, were this region involved in a more general process of rational choice (Kahnt & Tobler, 2013; Ni & Li, 2021; Zhang et al., 2017), we would have observed higher rejection rates for *fair*, *unfair*, and *no-alternative* offers during rTPJ stimulation. Analogously, if the rTPJ was involved in judgements of morality and fairness as elicited by the mini-UG task (Jeurissen et al., 2014; Young et al., 2010), we would have observed higher rates of rejections in response to both *unfair* and *no alternative* offers (not in response to *fair offers*). Therefore, the present data supported a crucial involvement of the rTPJ in the attribution of intention to the proposer when evaluating the context in which the proposal is made and, importantly, in evaluating proposer’s intentionality (Hao et al., 2023; Langenbach et al., 2022; Lee & Seo, 2016; Saxe & Wexler, 2005).

### Increase in rejections in nonbelievers in no-alternative offers

Moreover, we assessed the degree to which participants believed in the cover story and controlled for that in data analysis. When we compared the rejection rates between “believers” and “nonbelievers” by post-hoc exploratory analyses, we found a higher number of rejections in response to the *no alternative* offers in participants who did not believe in the cover story. Responses to *fair* and *unfair offers* did not differ between the two subgroups of participants. Although these results are explorative in their nature, they seem to support the interpretation of the main data. Indeed, believing to be in a trade with another agent is pivotal for activating mentalizing and possible mechanisms of intentionality attribution. We can speculate that only when participants interact with another real agent, they can attribute mental states and intentions (Abu-Akel et al., 2020; Özdem et al., 2017). Therefore, only when individuals can assume that the other agent had no better alternative to offer, they can decide to accept an offer although this is evaluated as unfair (i.e., *no alternative offers*). When no other agent is recognized on the opposite side, this mechanism is not activated, and no disposition to accept unfair offers is elicited. However, it is worth underlining that this interim interpretation should be considered as hypothesis-generating and requires specifically designed studies to be fully addressed.

These data could seem at odds with a previous seminal study by Sanfey et al. (2003) using the standard UG task, in which the participants accepted fair offers, with decreasing acceptance rates as the offers became less fair. Very unfair offers (i.e., offers of $2 and $1 compared with $5 and $3 in their setting) made by human partners were rejected at a significantly higher rate than the same offers made by a computer, suggesting that participants had a stronger emotional reaction to unfair offers from humans than to the same offers from a computer. However, in Sanfey et al.’s (2003) study, the participants were informed in advance that they would have been in touch with a human or a computer during different task phases, and this factor could have modulated the interaction differently. In our experimental design the participants were not told of the possibility of interacting with a computer. Therefore, the participants not believing in the cover story (“nonbelievers”) likely activated a different strategy aimed at maximizing their return without considering the other agent, just because they did not believe any agent to be involved in the interaction and no emotional reaction emerged (Wang et al., 2022). Moreover, as far as the mentalization process is concerned, the standard UG task used in Sanfey et al. (2003) is mostly used to target proposers’ ability to assess intentions of the other player, while it is not designed to target responders’ ability to evaluate partners’ intentions. For these reasons, our findings further supported an interpretation emphasizing a mechanism of perspective taking in responders’ when evaluating the bargains received. This interpretation of the data is in line with recent neuroimaging studies showing a modulation of activation of the rTPJ when participants ascribed intentions to nonhuman agents (Özdem et al., 2017). Moreover, this interpretation is consistent with evidence from a further study showing that interacting with humans (vs. computers) induced activations in a right lateralized subnetwork including the TPJ and the anterior paracingulate cortex, specifically contributing to spontaneous attribution of intentionality to human agents, within the wide mentalizing network (Abu-Akel et al., 2020). Overall findings of the present study nicely fit with a previous neuroimaging study using a poker paradigm in which the TPJ was demonstrated to be involved in socially guided decisions primarily involving human, i.e., highly relevant, agents (compared with computers; Carter et al., 2012).

### Refining evidence on the neural correlates of the UG task

The present study contributes to clarify the role of the rTPJ in the network sustaining social decision making in a context reproducing real-time interactions. Our data suggest that the rTPJ is involved in attribution of intentions to the other partner in the game, thus accepting or refusing the same type of offers depending on intentionality evaluation. Some previous studies (Blair-West et al., 2018; Speitel et al., 2019) have used noninvasive brain stimulation to identify the brain areas involved in the mentalization network activated by UG. In Blair-West et al.’s (2010) study, participants received anodal (vs. sham) transcranial direct current stimulation (tDCS) over the rTPJ before the standard UG task. No difference was found between anodal and sham stimulation on the total number of unfair offer rejections or RTs in the UG task. However, as stated by the authors themselves, the lack of significant differences could have been ascribed to methodological issues related to the tDCS protocol used and, importantly, to the experimental procedure that could not be sufficiently apt to offer a genuine experience of interaction with another person (Blair-West et al., 2018); as no debriefing was performed to screen participants who believed or not in the cover story, it was not possible to control for this factor in data analyses and interpretation of results. More recently, Speitel et al. (2019) used tDCS during the UG over the PFC or the rTPJ focusing on the roles of proposers as well as of responders. The results showed that stimulation of the rTPJ only affected the behavior in the proposer condition, leading to fairer offers, whereas no effect was found in responders. Complementarily, stimulation of the PFC increased the number of accepted offers in the role of responders only, with no effect in proposers. The lack of any effect of rTPJ stimulation in the role of responder in that study is probably due to the nature of the UG task used and corroborated our results using the mini-UG. Indeed, as it has been argued by Güroǧlu et al. (2010), the traditional version of the UG mainly allows to observe a reaction to basic fairness considerations in the responders, whereas the mini-UG specifically requires the responders to mentalize proposers’ intentions. The reason for the lack of any effect during stimulation of the rTPJ in the shoes of responders in Speitel et al.’s (2019) study is thus likely related to the fact that the traditional version of the UG was inefficient in targeting intention attribution in responders. Conversely, the UG task used by Speitel et al. (2019) was able to target intentions’ attribution in the shoes of proposers, as they had to assume the perspective of the other player to propose a valuable offer, resulting in significant modulation of performance after rTPJ stimulation.

### Considerations, limitations, and future directions

Our results and previous evidence identify some key regions involved in social decision making and refine previous evidence on the brain correlates of bargaining tasks. The results from the present study specifically supported a role of the rTPJ in mentalizing other’s intentions when evaluating unfair offers. This study, together with the previous ones on the same topic (Blair-West et al., 2018; Güroǧlu et al., 2010; Speitel et al., 2019), raises relevant methodological issues to be considered when investigating bargaining in experimental contexts. First, variations of the same experimental task can target different cognitive mechanisms in the partners involved. Second, when implementing experimental paradigms designed to reproduce social interactions in the laboratory, it is recommended to use a cover story to increase the feeling of being in touch with another agent and is pivotal to assess the extent to which participants trusted the proposed scenario. A third consideration refers to brain stimulation studies on this topic. One of the possible reasons previous studies could not obtain significant results was possibly related to the stimulation technique used (e.g., tDCS in the study from Blair-West et al., 2018), whereas the focal stimulation achieved by TMS probably allowed to overcome the spatial limitations of tDCS (Miniussi & Ruzzoli, 2013). Moreover, in previous studies multiple experimental sessions were needed to compare different stimulation conditions (active vs control), and the participants could have developed doubts about the cover story used, whereas we condensed the different stimulation conditions in a single session using an ABAB design. These aspects should be considered by future investigations on the same issue.

We acknowledge some limitations of the present study. A first set of observations deal with the use of the cover story. Although we tried to induce the feeling of being in interaction with another agent by using a cover story, a small part of the experimental sample disbelieved it. This is a major constraint in studies investigating behavior and cognition in experimental contexts reproducing real-life situations (Blair-West et al., 2018). Overall, our cover story worked reasonably well, but future works should contribute to develop experimental conditions ensuring complete adherence to the setting of social interaction and assess feasibility of cover stories better fitting the experimental paradigm at hand. Note that in the present experiment, the participants were informed that they would have received a gadget equivalent to the credits accumulated during the game but eventually received a gadget of the same value to avoid discriminating participants based on their response pattern. We were aware of the possible ethical implications of this choice; thus in the debriefing session, we ensured a thoughtful understanding of the experimental procedures by our participants, who did not raise any criticism (also because no mention was made in advance about the values of gadgets). Importantly, participants were asked not to disclose the experimental procedure to other individuals to limit full involvement of further participants. Another issue to be mentioned is that our experiment was not designed to assess the impact of the expected value of the gadget on participants’ responses during the task. Future studies could use gadgets of different values and assess the impact of their relative value on participants’ performance.

Our secondary analyses, including participants who did not believe in the cover story, allowed us to propose that participants adopted possibly different strategies if they believe to interact with a real agent versus if they do not. This finding was supported by a correlation analysis on the association between rejection rates of no alternative offers and extent of trustworthiness in the cover story in the whole group. Nonetheless, we want to acknowledge that the number of the “nonbeliever” participants was small and considerably smaller compared with the group of “believers,” thus limiting the strength of comparisons between the two groups. Moreover, it has to be stressed that our study was not strictly designed to compare participants in terms of their belief of being in interaction with other person, so that the present observations need to be confirmed by studies in which different experimental conditions will try to modulate participants’ expectations and the feeling of interacting with a real agent or with a predefined script presented by a computer. Interestingly, it would be worth investigating how behavioral responses, cognitive processes, and involvement of brain areas are modulated by engaging in bargaining with a human agent versus a virtual agent, considering the degree of intentionality attributed to them (Abu-Akel et al., 2020; Özdem et al., 2017).

Lastly, it is relevant to acknowledge that although the sample size was determined based on a priori power analysis, future studies could increase the number of participants, including both females and males, to replicate the present data and support the reliability of the protocol and to investigate whether gender affects social decision-making behavior and the corresponding brain correlates. Crucially, because our results are mainly based on a group-level analysis, it would be relevant to enroll larger samples of participants to investigate individual factors and latent variables that could influence social decision making, such as personality traits (Zhao & Smillie, 2015) and complex social emotional responses (Ramsøy et al., 2015).

## Conclusions

The present study supports an involvement of the rTPJ in intention attribution during bargaining. Our results contribute to clarify the processes involved during social interaction and enhance comprehension of TPJ contribution in complex social exchanges. In the future, to maximize the strength of the present findings and to address inconsistencies in previous works (Gaesser et al., 2019; Li et al., 2020), it would be relevant to study such complex dynamics in more ecological contexts, for example combining brain stimulation and neuroimaging techniques in a face-to-face interaction in the same experimental design (Panico et al., 2021). This could improve ecological validity of the findings while simultaneously targeting the brain networks involved in social cognition (Van Overwalle & Mariën, 2016).

### Supplementary Information

Below is the link to the electronic supplementary material.

Supplementary file1 (DOCX 159 KB)

## Data Availability

Data will be provided upon reasonable request.

## Code availability

The script of the experimental task will be provided upon reasonable request.

## Declarations

### Open practice statement

Data or materials for the experiments are available upon request, and none of the experiments was preregistered.
