# Supplementary material for: The involvement of rTPJ in intention attribution during social decision making: A TMS study
Source: Cogn Affect Behav Neurosci. 2024 Apr 30;24(4):755–65. doi: 10.3758/s13415-024-01188-7 (PMC11233285; doi:10.3758/s13415-024-01188-7)
Supplement: Supplementary file 1 — Supplementary file1 (DOCX 159 KB) [file 13415_2024_1188_MOESM1_ESM.docx]

**Supplementary Materials**

**The involvement of rTPJ in intention attribution during social decision making: a TMS study**

Francesco Panico, Antonella Ferrara, Laura Sagliano, Luigi Trojano

University of Campania “Luigi Vanvitelli”, Viale Ellittico 31, 81100 Caserta, Italy

**Post Experimental Questionnaire.** Participants were asked to fill in the form at the end of the experimental session (items translated into English language).

Please answer the question using a 5-point scale (*from 1= “not at all” to 5= “completely”*):

- How energic do you feel?
- How tense do you feel?
- How sleepy do you feel?
- How pleasant was the interaction with the other player?
- How much did you feel like you were playing with another player?

**Bayesian Analyses**. Results from the Bayesian Wilcoxon Signed-Rank Test comparing the different offer types (alt-fair, alt-unfair, no-alt) during stimulation (rTPJ-TMS, Vertex-TMS)

| Measure 1 | |  | | Measure 2 | | BF₁₀ | | W | | Rhat | |
| --- | --- | --- | --- | --- | --- | --- | --- | --- | --- | --- | --- |
| rTPJ-TMS_alt-fair |  |  |  | Vertex-TMS_alt-fair |  | 0.27 |  | 12.50 |  | 1.00 |  |
| rTPJ-TMS_alt-unfair |  |  |  | Vertex-TMS_alt-unfair |  | 0.46 |  | 55.00 |  | 1.00 |  |
| rTPJ-TMS_no-alt |  |  |  | Vertex-TMS_no-alt |  | 11.62 |  | 92.00 |  | 1.00 |  |
|  | | | | | | | | | | | |
| *Note.*  Result based on data augmentation algorithm with 5 chains of 1000 iterations. | | | | | | | | | | | |

**
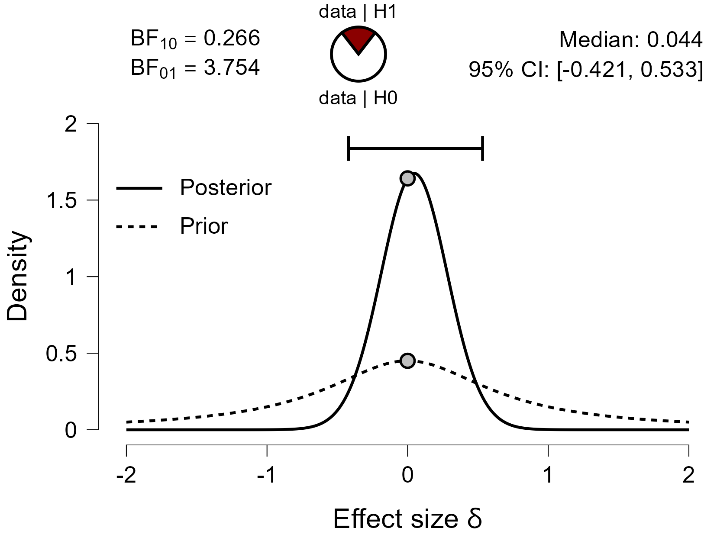

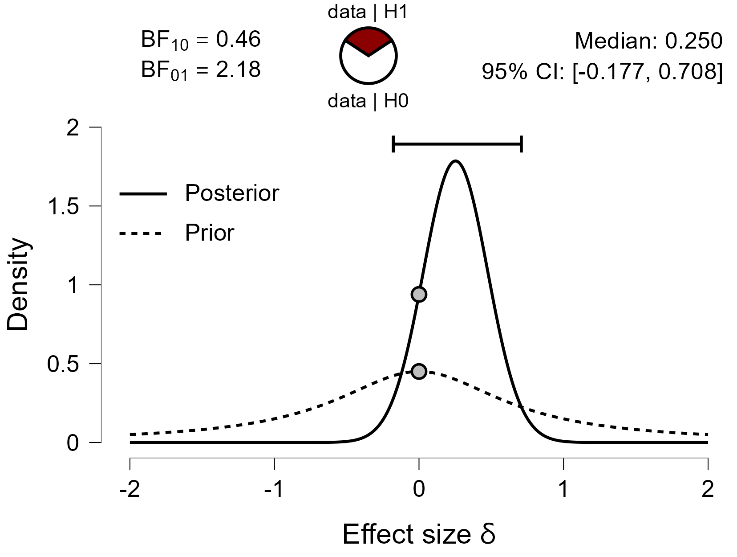
**

**
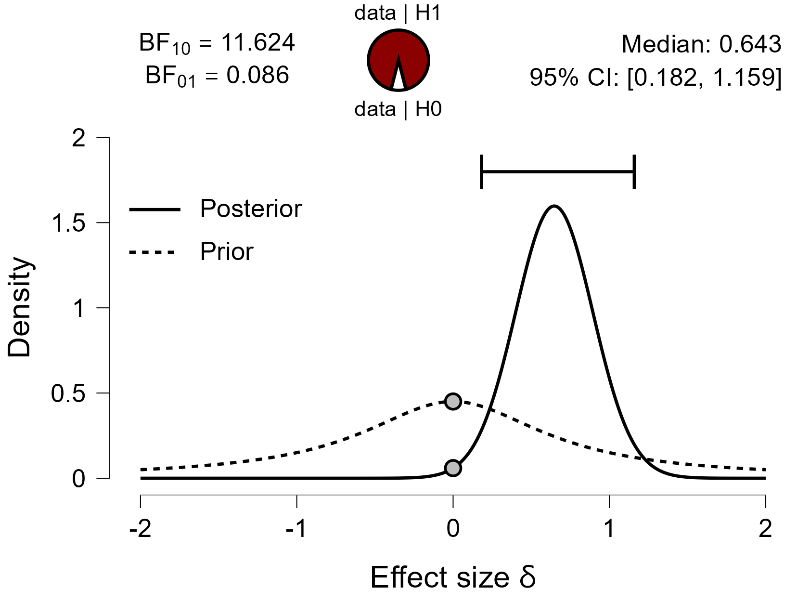
**

**Prior and posterior plots**. The gray dots indicate the prior and posterior density at the test value (Dickey & Lientz, 1970; Wagenmakers et al., 2010) for the comparison of *alt-fair offers* (top left), *alt-unfair offers* (top right) and *no-alt* *offers* (bottom) under rTPJ-TMS and Vertex-TMS.

**Supplementary Table 1.** Results from Mann-Whitney tests on the rejections in the two subgroups (believers and non-believers) for the stimulation conditions (rTPJ-TMS and Vertex-TMS) and the different trials (alt-fair, alt-unfair, and no-alt)

| Offer/Stimulation | Believers (n=19)  Mean (SD) | Non-believers (n=7)  Mean (SD) | Mann-Whitney U | p-value | Effect size (r) |
| --- | --- | --- | --- | --- | --- |
| rTPJ-TMS_alt-fair | .63 (1.53) | .43 (.79) | 69.50 | .86 | .05 |
| rTPJ-TMS_alt-unfair | 16.32 (5.97) | 19.00 (2.24) | 85.50 | .28 | .23 |
| rTPJ-TMS_no-alt | 12.26 (6.82) | 17.14 (3.67) | 101.00 | **.048** | .39 |
| Vertex-TMS_alt-fair | .53 (1.84) | .14 (.38) | 65.00 | .95 | .03 |
| Vertex-TMS_alt-unfair | 15.53 (6.20) | 18.57 (1.27) | 75.00 | .65 | .10 |
| Vertex-TMS_no-alt | 10.47 (7.73) | 17.57 (3.60) | 105.00 | **.025** | .44 |

| Variable | 1 | 2 | 3 | 4 | 5 | 6 | 7 |
| --- | --- | --- | --- | --- | --- | --- | --- |
| 1. Trustworthiness to cover story  (*1-5)* | - |  |  |  |  |  |  |
| 2. Rejections rTPJ-TMS_alt-fair *(0-20)* | -.13 | - |  |  |  |  |  |
| 3. Rejections rTPJ-TMS_alt-unfair *(0-20)* | -.07 | -.28 | - |  |  |  |  |
| 4. Rejections rTPJ-TMS_no-alt *(0-20)* | -.36 | .09 | **.64**** | - |  |  |  |
| 5. Rejections Vertex-TMS_alt-fair *(0-20)* | .14 | .10 | .14 | .28 | - |  |  |
| 6. Rejections Vertex-TMS_alt-unfair *(0-20)* | -.02 | -.12 | **.62**** | .**61**** | .002 | - |  |
| 7. Rejections Vertex-TMS_no-alt *(0-20)* | **-.45*** | .03 | **.55**** | **.89**** | .06 | **.61**** | - |

**Supplementary Table 2**. Results from Spearman correlations to look for associations between the degree of belief in the cover story (1-5 scale) and the number of rejections of each offer under rTPJ and vertex stimulation on the whole sample (n=26)

*=p<.05;

**=p<.01;

**Supplementary Table 3.** Results from Mann-Whitney tests on RTs in the two subgroups (believers and non-believers) for the stimulation conditions (rTPJ-TMS and Vertex-TMS) and the different trials (alt-fair, alt-unfair, and no-alt)

| Offer/Stimulation | Believers (n=19)  Mean (SD) | Non-believers (n=7)  Mean (SD) | Mann-Whitney U | p-value | Effect size (r) |
| --- | --- | --- | --- | --- | --- |
| rTPJ-TMS_alt-fair | 481.89 (295.07) | 323.20 (196.85) | 43.00 | .19 | .27 |
| rTPJ-TMS_alt-unfair | 531.77 (297.68) | 384.29 (261.39) | 41.00 | .15 | .29 |
| rTPJ-TMS_no-alt | 580.94 (305.73) | 423.26 (302.28) | 39.00 | .12 | .31 |
| Vertex-TMS_alt-fair | 467.12 (249.86) | 351.86 (109.66) | 53.00 | .46 | .15 |
| Vertex-TMS_alt-unfair | 565.98 (354.96) | 421.21 (256.06) | 53.00 | .46 | .15 |
| Vertex-TMS_no-alt | 537.83 (272.86) | 387.40 (288.47) | 42.00 | .17 | .28 |

**Supplementary Table 4.** Results from Mann-Whitney tests on number of rejections in the two subgroups (Group 1 receiving Vertex stimulation *after* TPJ stimulation; and Group 2 receiving Vertex stimulation *before* TPJ stimulation) for the different trials (alt-fair, alt-unfair, and no-alt)

| Offer/Stimulation | Group 1 (n=11)  Mean (SD) | Group 2 (n=8)  Mean (SD) | Mann-Whitney U | p-value | Effect size (r) |
| --- | --- | --- | --- | --- | --- |
| Vertex-TMS_alt-fair | .00 (.00) | .63 (1.06) | 60.50 | .18 | .49 |
| Vertex-TMS_alt-unfair | 8.09 (3.36) | 6.75 (3.19) | 34.50 | .44 | .19 |
| Vertex-TMS_no-alt | 5.45 (4.28) | 3.75 (3.19) | 31.00 | .31 | .25 |

**Supplementary Table 5.** Results from Mann-Whitney tests on RTs in the two subgroups (Group 1 receiving Vertex stimulation *after* TPJ stimulation; and Group 2 receiving Vertex stimulation *before* TPJ stimulation) for the different trials (alt-fair, alt-unfair, and no-alt)

| Offer/Stimulation | Group 1 (n=11)  Mean (SD) | Group 2 (n=8)  Mean (SD) | Mann-Whitney U | p-value | Effect size (r) |
| --- | --- | --- | --- | --- | --- |
| Vertex-TMS_alt-fair | 457.27 (243.19) | 595.10 (393.70) | 49.00 | .72 | .09 |
| Vertex-TMS_alt-unfair | 540.80 (218.53) | 818.08 (555.68) | 52.00 | .55 | .15 |
| Vertex-TMS_no-alt | 521.77 (245.76) | 867.70 (490.83) | 61.00 | .18 | .32 |
